# Clinical and Acoustic Alterations of Swallowing in Children Exposed to Zika Virus during Pregnancy in a Cohort in Amazonas, Brazil: A Case Series Study

**DOI:** 10.3390/v15122363

**Published:** 2023-11-30

**Authors:** Cristina de Souza Rodrigues, Raillon Keven Santos Souza, Cosmo Vieira Rocha Neto, Rodrigo Haruo Otani, Daniel de Medeiros Batista, Ana Karla Nelson de Oliveira Maia, Kleber Pinheiro de Oliveira Filho, Thais Dourado de Andrade, Emmilyn de Andrade Almeida, Luiz Henrique Gonçalves Maciel, Lucíola de Fátima Albuquerque Almeida Peixoto Castro, Marília Rosa Abtibol-Bernardino, Djane Clarys Baia-da-Silva, Silvana Gomes Benzecry, Marcia da Costa Castilho, Flor Ernestina Martínez-Espinosa, Maria das Graças Costa Alecrim, Rosane Sampaio Santos, Camila Botto-Menezes

**Affiliations:** 1 Postgraduate Program in Tropical Medicine (PPGMT), University of Amazonas State (UEA), Manaus 69040-000, Brazil; fgacristinarodrigues@gmail.com (C.d.S.R.); luizmaciel.lh@gmail.com (L.H.G.M.); peixoto.luciola@gmail.com (L.d.F.A.A.P.C.); marilia.abtibol@gmail.com (M.R.A.-B.); florespinosa@gmail.com (F.E.M.-E.); galecrim.br@gmail.com (M.d.G.C.A.); 2 Northern University Center (UniNorte), Manaus 69020-160, Brazil; raillonkevensantos@gmail.com; 3 School of Health Sciences, University of Amazonas State, Manaus 69065-001, Brazil; vieiracosmo93@gmail.com (C.V.-R.N.); rod.otani@gmail.com (R.H.O.); dmb.med@uea.edu.br (D.d.M.B.); aknom.med@uea.edu.br (A.K.N.d.O.M.); sbenzecry@uea.edu.br (S.G.B.); 4 Faculty of Pharmacy, University Nilton Lins, Manaus 69058-030, Brazil; kleberpinheiro2@icloud.com (K.P.d.O.F.); tddandrade@gmail.com (T.D.d.A.); 5 East Zone Children’s Emergency Hospital, Manaus 69058-030, Brazil; emmilyn.fono@gmail.com; 6 Department of Maternal and Child Health, Medical School, Federal University of Amazonas, Manaus 69020-160, Brazil; 7 Leônidas & Maria Deane Institute, Fiocruz Amazonia, Manaus 69057-070, Brazil; 8 Tropical Medicine Foundation Doutor Heitor Vieira Dourado (FMT-HVD), Manaus 69040-000, Brazil; mcastilho@fmt.am.gov.br; 9 Medical Course Coordination at Manaus Metropolitan College/FAMETRO, Manaus 69050-000, Brazil; 10 Postgraduate Program in Communication Disorders (PPGDIC), University of Tuiuti do Paraná (UTP), Paraná 82010-210, Brazil; rosane.santos2@utp.br

**Keywords:** *Zika virus*, arbovirus, congenital *Zika virus* syndrome, oropharyngeal dysphagia, non-microcephalic children

## Abstract

Oropharyngeal dysphagia (OD) is a swallowing disorder that involves difficulty in safely passing the food bolus from the oral cavity to the stomach. OD is a common problem in children with congenital Zika virus syndrome (CZS). In this case series, we describe the clinical and acoustic alterations of swallowing in children exposed to the Zika virus during pregnancy in a cohort from Amazonas, Brazil. From July 2019 to January 2020, 22 children were evaluated, 6 with microcephaly and 16 without microcephaly. The mean age among the participants was 35 months (±4.6 months). All children with microcephaly had alterations in oral motricity, mainly in the lips and cheeks. Other alterations were in vocal quality, hard palate, and soft palate. Half of the children with microcephaly showed changes in cervical auscultation during breast milk swallowing. In children without microcephaly, the most frequently observed alteration was in lip motricity, but alterations in auscultation during the swallowing of breast milk were not observed. Regarding swallowing food of a liquid and pasty consistency, the most frequent alterations were incomplete verbal closure, increased oral transit time, inadequacy in capturing the spoon, anterior labial leakage, and increased oral transit time. Although these events are more frequent in microcephalic children, they can also be seen in non-microcephalic children, which points to the need for an indistinct evaluation of children exposed in utero to ZIKV.

## 1. Introduction

Zika virus (ZIKV) is a flavivirus arbovirus transmitted primarily through the bite of female Aedes mosquitoes [1]. It was first identified in Uganda, Africa, in 1947, with its first epidemic occurring on Yap Island in Micronesia; however, there have been previous reports of infection in humans in other countries in Africa and Asia [2,3,4]. ZIKV was identified in the Americas, more specifically in Brazil, at the end of 2015, although phylogenetic studies have identified the arrival to have been in mid-2013 [5,6,7]. In Brazil, the high number of cases of microcephaly in children exposed in utero to ZIKV in 2015 evidenced the important association and the need for multidisciplinary follow-up of pregnant women exposed to ZIKV and their children [8,9,10]. ZIKV infection during pregnancy, especially in the first and second trimesters of pregnancy, can trigger damage to the fetus’s central nervous system, especially during embryonic development, resulting in impactful characteristics for the child [11,12]. The main implications of intrauterine exposure are complications from congenital Zika virus syndrome (CZVS), changes in growth and development, and low birth weight, in addition to speech and swallowing problems [13,14,15,16,17,18,19].

Oral and phonological impairments are associated with microcephaly induced by ZIKV and include bruxism, mixed breathing, changes in muscle tone that compromise the swallowing process, hearing loss, dysphagia, altered tongue frenulum, and delays in neuropsychomotor and language development [19,20,21,22,23,24,25]. Oropharyngeal dysphagia (OD) is a swallowing disorder that involves difficulty in safely passing the food bolus from the oral cavity to the esophagus [26,27]. OD can be caused by neurological factors arising from congenital abnormalities or combined with damage associated with diseases of the oral cavity, pharynx, and upper esophageal sphincter [28,29]. OD can lead to complications such as aspiration pneumonia, dehydration, and malnutrition associated with inadequate nutritional intake [30]. Among the typical signs and symptoms of OD are dysfunction of the labial or facial muscles, inability to chew or push food, xerostomia, sialorrhea, difficulty in initiating swallowing, nasal regurgitation, the need for several swallows, coughing, and wet voice during and after meals [30,31,32]. It is necessary to identify which phase or which phases of swallowing are compromised (oral, pharyngeal, or esophageal phases), as well as the etiology (stroke, head injury, dementia, Parkinson’s disease, cancer, multiple sclerosis, myasthenia gravis, dermatomyositis, complicated reflux, or large hiatal hernias) and degree of dysphagia (mild, moderate, or severe) [29,33].

In ZIKV-related dysphagia, mostly associated with microcephaly, the child has difficulty in managing liquid consistency, due to changes in facial muscles, decreased muscle tone, and intraoral hyposensitivity, which causes oral leakage through the labial commissures and silent aspiration [34,35]. This study describes the clinical and acoustic characteristics of the swallowing of children exposed to ZIKV during pregnancy and cared for in a tertiary unit, to serve as a reference for the diagnosis and treatment of tropical and infectious diseases in the Amazon.

## 2. Materials and Methods

Children born between March 2016 and June 2018 from mothers studied in previous cohorts [17,36,37] were clinically evaluable in terms of their swallowing ability. Mothers’ information was obtained from electronic medical records. The ZIKV infection in the mother was confirmed in a blood or urine sample through real-time reverse transcriptase polymerase chain reaction (RT-PCR) detection. RT-PCR was performed following the protocol of Lanciotti et al. [38] at the Central Public Health Laboratory in Amazonas (LACEN-AM). Tests for Dengue-virus, Parvovirus-B19-virus infections, and the detection of etiological agents of TORCH Syndrome and malaria were performed by the Virology Laboratory and Clinical Analysis Laboratory at the Tropical Medicine Foundation Doutor Heitor Vieira Dourado (FMT-HVD), as previously described [17]. These infections were evaluated because they can induce embryonic malformations.

The clinical and laboratory variables of the children during birth are summarized in Table 1. The children’s eating habits were obtained through interviews with the parents. Children were not evaluated for ZIKV infection. The clinical assessment was performed using the pediatric dysphagia clinical evaluation protocol 2014 (PAD-PED) [39]. The development of the stomatognathic system was carried out according to the previously described methodology [40]. Posture, tone/strength, structure, and/or motility of the lips, tongue, cheek, and palate were evaluated (Table 2). Vocal quality, mucosa aspect, frequency of saliva swallowing, and cervical auscultation were also evaluated. Swallowing in the oral and pharyngeal phases was primarily assessed using foods provided by the Nutrition Department of the FMT-HVD, and the selection of consistencies respected the participant’s food introduction (Table 3). All clinical and swallowing assessments were performed by an experienced speech therapist.

The acoustic analysis of swallowing was performed using a portable Doppler sonar (DS) device (wave angle sound: 3 MHz). This equipment was coupled to the notebook software and the acoustic sounds were recorded and later analyzed using Deglutisom software^®^ version 2018.02.07. The transducer was positioned on the right side of the neck, in the lateral portion of the trachea, just below the cricoid cartilage. To reduce dispersion and advance the sound recording, conductive gel was used. The instrument used to collect acoustic information was the Acoustic Swallowing Assessment Protocol (PAAD) [41]. OD was defined as an alteration in the clinical evaluation of structural and functional features, evaluation of saliva swallowing, evaluation of non-nutritive sucking, and evaluation with food, and/or the presence of alterations in the acoustic signs of swallowing.

OD was defined and classified based on PAD-PED and Oliveira et al.’s work [40], as absent (no abnormalities), mild (presence of impaired oropharyngeal transit, but without signs of aspiration), moderate (i.e., impaired oropharyngeal transit, with the presence of aspiration signs and preserved protection mechanisms, allowing clearance of the lower airways), and serious (i.e., compromised oropharyngeal transit, with signs suggestive of aspiration and an absence of protective mechanisms).

This study was approved by the FMT-HVD Research Ethics Committee, being assigned ethical approval number 08941019.2.0000.0005/2019. All guardians of the participants provided formal written consent. The variables of interest in this study were registered in a standardized questionnaire using Epi Info software, version 7. Data analyses were carried out in the Stata program, version 13. The results were expressed through relative frequencies, with mean and standard deviation.

## 3. Results

Table 1 expresses the baseline characteristics of children exposed to ZIKV infection in the intrauterine period in Manaus, Amazonas, Brazil. From July 2019 to January 2020, 22 children were evaluated, 6 with microcephaly (cases 4, 6, 7, 8, 15, and 19) during the assessment and 17 without microcephaly. Five children were microcephalic at birth (cases 4, 6, 8, 15, and 19). In other words, case 7 is classified as postnatal microcephaly, although the mother had a confirmed ZIKV infection. The neurological characteristics of three microcephalic children are described in Supplementary Table S1 (cases 4, 7, and 19). The mean gestational and enrollment ages were 38.9 weeks (±0.86 weeks) and 35 months (±4.6 months), respectively. The children averaged 2.99 kg (±0.44 kg) at birth. The neonatal hearing screening was not suitable for one child, while the tongue test was altered for one child (7.1%, case 1). Cardiac and/or respiratory problems were seen in three children (cases 4, 5, and 11). No mother tested positive for Dengue-virus, Parvovirus-B19-virus infections, etiological agents of TORCH Syndrome, or malaria during the pregnancy.

Changes in oral motricity and the frequency of saliva swallowing through structural and functional evaluation are shown in Table 2. All children with microcephaly presented alterations in oral motricity, mainly in the lips and cheeks (100%, 6/6), hard palate (cases 6, 7, and 8), soft palate (cases 6, 8, and 15), and vocal quality (cases 7, 8, 15, and 19). In children without microcephaly, the most frequently observed alteration was in lip motricity, seen in 43.7% (7/16). In both groups, the appearance of the oral mucosa was adequate (100%, 22/22). In terms of the frequency of saliva swallowing, children with microcephaly mostly had sialorrhea (50%, cases 6, 8, and 19) and sialostasis (33.3%, cases 7 and 15). In terms of cervical auscultation, 50% of the children with microcephaly (cases 8, 15, and 19) had alterations after swallowing. In conclusion, three cases of mild oropharyngeal dysphagia, two cases of moderate to severe oropharyngeal dysphagia, and one case of severe oropharyngeal dysphagia were evidenced.

The children with microcephaly showed significant changes in swallowing and swallowing acoustics (Table 3 and Table 4). When swallowing food in liquid consistency, children with microcephaly presented incomplete verbal restraint (80%, 4/5, cases 4, 6, 15, and 19) and increased oral transit time (60%, 3/5), while when swallowing food with a pasty consistency, inadequacy in capturing the spoon was seen in 80% (4/5), with anterior labial leakage in 60% (3/5), and increased oral transit time in 60% (3/5). In addition, children with microcephaly had an average time increase of 1.11 s and an average intensity decrease of 51.58 dB. Children without microcephaly had no alterations.

Children with oropharyngeal dysphagia presented with microcephaly (*p* < 0.001), inadequate frequency of swallowing (0.009), and altered cervical auscultation (0.013) (Table 4).

## 4. Discussion

ZIKV infection in pregnancy is a concern as it is linked to catastrophic fetal abnormalities, including microcephaly, miscarriage, intrauterine growth restriction, changes in growth and development, low birth weight, growth velocity, and swallowing issues [13,14,15,16,17,18,19]. In this study, we have described the swallowing assessment of 22 children exposed intrauterine to ZIKV based on a standardized protocol and assessments and using DS. PAD-PED is a standardized protocol, developed by the Pontifical Catholic University of São Paulo, used for the clinical evaluation of OD in children, is widely used in Brazilian studies, and incorporates information provided by caregivers and clinical assessment, in addition to including an assessment of muscle tone, posture, and mobility of the stomatognathic system and a functional assessment of swallowing [39]. SD is a non-invasive, painless, low-cost method that does not expose the patient to radiation and is promising among methods for evaluating swallowing in adults, children, and babies [42,43]. SD is based on the assessment of swallowing sounds and audible cues and provides a reliable classification for screening and identifying patients with a higher risk of aspiration and laryngeal penetration [44,45]. Using these techniques, we evidenced six children with OD, across different classifications, and all of the children had microcephaly (100%).

Alterations in orofacial motricity were evidenced both in children with microcephaly and in those without microcephaly. The unanimous characteristics found in the population with microcephaly during the motricity evaluation were parted lips, tongue mobility, and decreased cheek tone. The presence of sialorrhea and sialostasis in part of the population with microcephaly was also verified, as well as the presence of a wet voice and altered cervical auscultation during voluntary swallowing. Oliveira et al. [40] showed similar aspects when evaluating 116 children (58 children exposed to Zika virus without microcephaly and 58 children with microcephaly related to Zika virus), with microcephalic children being more likely to have an inadequate resting posture for feeding, abnormality related to movement and tonus of the stomatognathic system, and OD. In the present study, all children with microcephaly presented OD.

The functional evaluation of swallowing foods with liquid consistencies showed incomplete lip sealing and increased oral transit time, while with pasty consistencies, inadequate grasping of the spoon, increased oral transit time, increased mean swallowing time, and reduced mean intensity were seen mainly in microcephalic children. Such events may be associated with inadequate neuromuscular skills and neurological damage of cortical origin in children with microcephaly [46,47]. A previous study carried out with children exposed to ZIKV showed that four microcephalic children (two of which are part of this case description, case 4 and 19, Supplementary Table S1) presented important neurological changes that were reflected in delays in neuropsychomotor development (delays in cognitive, language, motor, and psychosocial abilities), spastic tetraparesis, changes in imaging examination (deficits in social interaction, deficits in muscle strength, muscle tone abnormalities, hyperreflexia, osteotendinous, cranial nerve abnormalities, and epilepsy), and neuroimaging exams (with cerebral calcifications, ventricular dilatation, lissencephaly/pachygyria, cortico-subcortical atrophy, megacisterna magna, and periventricular leukomalacia) [37]. These changes in themselves are related to the inadequate transit of the food bolus from the mouth to the esophagus, due to an abnormality, generally cerebral or muscular. The involvement of the neuronal network in the cortical region and brain stem can leave the individual susceptible to OD in the oral and pharyngeal phases of swallowing [48,49].

OD has been demonstrated in individuals with spastic tetraparesis [50,51,52], mild cognitive impairment, neurological deficits, and cranial nerve abnormalities [53,54,55,56], in children with speech and language delays [57], and in diseases with reflexes and motor delay [58], muscle tone abnormalities, and deficits in muscle strength [59]. In addition, impairments of motor function and coordination of tongue movements cause a reduction in ejection pressure and the impairment of effective swallowing, leading to an accumulation of food in the oral and pharyngeal cavity, responsible for cervical sound changes during and after swallowing [60].

One child (case 7), in this series of cases, had a normal head circumference at birth and microcephaly during evaluation and other follow-up visits. This child had neurological and swallowing functional inadequacy. This child was exposed in utero; however, we did not evaluate factors (including genetics, perinatal injury, and postnatal injury) that could also have justified postnatal microcephaly. In general, the prognosis is worse for children who have had an intrauterine infection or have a chromosomal or metabolic abnormality [61].

OD induced by ZIKV affects growth and psychomotor development, leading to important deleterious effects on child development, especially related to growth and weight [62]. In the present study, we did not evaluate the children’s weight during the speech–language pathology assessment. However, one cohort, which included much of the sample described here (Supplementary Table S2), was described to show growth velocity (GV) and nutritional status based on World Health Organization standards of children exposed to ZIKV during pregnancy [17]. A similar frequency of low GV was shown across microcephalic and non-microcephalic children. Furthermore, the authors showed that children with changes in GV showed changes in neurological exams, although these were not statistically significant, when compared to children with adequate GV. Neurological changes were related to OD.

In this study, it was not possible to perform acoustic analyses of swallowing foods of all consistencies, due to acceptance of the food, non-introduced consistencies, and the user using an alternative feeding route. The average intensity in the liquid, pasty, and solid consistency was below expectations, demonstrating the decrease in muscle strength during the swallowing process; furthermore, in foods with a pasty consistency, the oral transit time was increased with a greater impact on children with microcephaly, correlating with the findings of clinical speech therapy assessment.

This study has limitations that are inherent of case reports. For example, in this series of cases, we only evidenced disorders in the oral and pharyngeal phases of swallowing due to the use of a non-invasive method. The use of invasive methods, such as videofluoroscopy and fiberoptic endoscopic swallowing, are more effective for exploring the dynamics, changes, safety, and efficacy of swallowing, as well as selecting and evaluating specific therapeutic strategies [63]. However, through the pediatric dysphagia clinical evaluation protocol 2014 and acoustic analysis with DS, two simple methodologies, we highlighted OD and its degrees of involvement. Due to the ease of implementation, these two methodologies can guide the first Rehabilitation for Swallowing Disorder procedures, especially in places that lack the necessary equipment for the diagnosis of OD or are far from large health units, or when the patient is unable to travel. Another limitation is that the children were not evaluated when positive for ZIKV infection, and therefore we could not define the etiology of the ZIKV in a patient who presented postnatal microcephaly.

## 5. Conclusions

The findings of this study are important in showing that oral motor dysfunction can be a factor associated with reduced food intake, increased energy expenditure, risk of malnutrition, choking, broncho aspiration, and aspiration pneumonia, especially in a group of children with microcephaly. Thus, longitudinal follow-up of children exposed to ZIKV infection in the intrauterine period is necessary for providing information for the correct understanding and management of swallowing difficulties, as well as other resulting problems.

## Figures and Tables

**Table 1:** Baseline characteristics of children exposed to the Zika virus in intrauterine and assessment by a speech therapist in Manaus, Amazonas, Brazil.

ID	Sex	Age *	Gestational Age (w)	Birth Weight (Kg)	Apgar	NHS	Tongue Test	Breathing and Heart Problems
1	M	35	40	3.13	9	Adequate	Changed	No
2	M	39	39	2.10	9	Adequate	Adequate	No
3	F	38	39	3.60	9	Adequate	ND	No
4	M	37	39	2.71	9	Adequate	ND	Cardiac
5	M	32	40	3.05	9	Adequate	Adequate	Respiratory
6	M	35	39	2.92	9	Adequate	Adequate	No
7	M	41	39	3.17	9	ND	ND	No
8	M	39	39	2.65	8	Not suitable	Adequate	No
9	F	30	39	3.15	9	Adequate	Adequate	No
10	M	40	40	3.40	8	Adequate	ND	No
11	M	33	38	2.74	9	Adequate	Adequate	Respiratory and cardiac
12	M	34	36	3.29	9	Adequate	Adequate	No
13	F	36	40	3.87	9	Adequate	Adequate	No
14	F	33	38	3.10	9	Adequate	Adequate	No
15	F	23	39	2.75	8	ND	ND	No
16	F	37	39	2.23	9	Adequate	Adequate	No
17	F	35	39	2.51	9	Adequate	ND	No
18	M	32	39	3.65	9	Adequate	Adequate	No
19	M	37	38	2.83	9	Adequate	Adequate	No
20	M	27	39		9	Adequate	ND	No
21	F	41	39	2.73	8	Adequate	Adequate	No
22	M	41	39	3.26	9	Adequate	ND	No

* Age at time of assessment in months, ID: identification, M: male, F: female, W: weeks, Cm: centimeters, NHS: neonatal hearing screening, and ND: not determined. Microcephalic patients are marked in gray.

**Table 2:** Changes in orofacial motricity and saliva through clinical evaluation speech therapy (structural and functional examination) in children exposed to the Zika virus in the intrauterine period in Manaus, Amazonas, Brazil.

	Lips	Tongue	Cheek	Palate	Vocal Quality	Frequency of Swallowing	Cervical Auscultation	OD Classification
1	Parted, tone decreased, and mobility altered	Posture and tone adequate, mobility not rated	Adequate	Hard inadequate and soft adequate	Adequate	Adequate	Adequate	Absent
2	Occluded, tone and mobility adequate	Posture and tone adequate, mobility not rated	Adequate	Hard inadequate and soft adequate	Adequate	Adequate	Adequate	Absent
3	Occluded, tone adequate, and mobility altered	Posture altered, tone and mobility adequate	Adequate	Hard suitable and soft adequate	Adequate	Adequate	Adequate	Absent
4	Parted, tone and mobility adequate	Posture and tone adequate, mobility not rated	Adequate	Hard suitable and soft adequate	Adequate	Adequate	Adequate	Mild
5	Occluded, tone and mobility adequate	Posture, tone, and mobility adequate	Adequate	Hard suitable and soft adequate	Adequate	Adequate	Adequate	Absent
6	Parted, tone and mobility altered	Posture and tone altered, mobility not rated	Reduced	Hard and soft inadequate	Adequate	Sialorrhea	Adequate	Moderate
7	Parted, tone and mobility altered	Posture and tone altered, mobility not rated	Reduced	Hard inadequate and soft adequate	Inadequate	Sialostasis	Adequate	Mild
8	Parted, tone and mobility adequate	Posture and tone altered, mobility not rated	Reduced	Hard and soft inadequate	Inadequate	Sialorrhea	Altered (worsened after swallowing)	Serious
9	Occluded, tone and mobility adequate	Posture, tone, and mobility adequate	Adequate	Hard suitable and soft adequate	Adequate	Adequate	Adequate	Absent
10	Parted, tone altered, and mobility adequate	Posture, tone, and mobility adequate	Reduced	Hard suitable and soft adequate	Adequate	Adequate	Adequate	Absent
11	Parted, tone and mobility adequate	Posture and tone adequate, mobility not rated	Adequate	Hard suitable and soft adequate	Adequate	Adequate	Adequate	Absent
12	Occluded, tone and mobility adequate	Posture and tone adequate, mobility not rated	Adequate	Hard suitable and soft adequate	Adequate	Adequate	Adequate	Absent
13	Occluded, tone and mobility adequate	Posture, tone, and mobility adequate	Adequate	Hard suitable and soft adequate	Adequate	Adequate	Adequate	Absent
14	Parted, tone and mobility altered	Posture and tone altered, mobility adequate	Adequate	Hard inadequate and soft adequate	Adequate	Adequate	Adequate	Absent
15	Parted, tone and mobility altered	Posture and tone altered, mobility not rated	Reduced	Hard suitable and soft inadequate	Inadequate	Sialostasis	Altered (worsened after swallowing)	Moderate
16	Parted, tone altered, and mobility adequate	Posture and tone altered, mobility not rated	Adequate	Hard suitable and soft adequate	Adequate	Adequate	Adequate	Absent
17	Parted, tone decreased, and mobility adequate	Posture and tone altered, mobility not rated	Reduced	Hard suitable and soft adequate	Inadequate	Adequate	Adequate	Absent
18	Parted, tone and mobility adequate	Posture, tone, and mobility adequate	Adequate	Hard suitable and soft adequate	Adequate	Adequate	Adequate	Absent
19	Parted, tone and mobility adequate	Posture and tone altered, mobility not rated	Reduced	Hard suitable and soft adequate	Inadequate	sialorrhea	Altered (worsened after swallowing)	Mild
20	Occluded, tone and mobility adequate	Posture, tone, and mobility adequate	Adequate	Hard suitable and soft adequate	Adequate	Adequate	Adequate	Absent
21	Occluded, tone and mobility adequate	Posture, tone, and mobility adequate	Adequate	Hard suitable and soft adequate	Adequate	Adequate	Adequate	Absent
22	Occluded, tone and mobility adequate	Posture, tone, and mobility adequate	Adequate	Hard suitable and soft adequate	Adequate	Adequate	Adequate	Absent

ID: identification. OD: oropharyngeal dysphagia. Microcephalic patients are marked in gray.

**Table 3:** Swallowing assessment according to food consistency in children exposed to Zika virus in the intrauterine period in Manaus, Amazonas, Brazil.

			Liquid							Pasty						Solid		
ID	Lip Seal	Oral Transit Time	Laryngeal Elevation	Average Time	Average Frequency (Hz)	Average Loudness (dB)	Lip Seal	Oral Transit Time	Laryngeal Elevation	Average Time (s)	Average Frequency (Hz)	Average Loudness (dB)	Lip Seal	Oral Transit Time	Laryngeal Elevation	Average Time (s)	Average Frequency (Hz)	Average Loudness (dB)
1	Adequate	Adequate	Present	1.2	1027	5.6	NA	NA	NA	NR	NA	NA	NA	NA	NA	NA	NA	NA
2	Adequate	Adequate	Present	7	1270	7	Adequate	Adequate	Present	0.5	1410	12	NA	NA	NA	NA	NA	NA
3	Adequate	Adequate	Present	1.5	1308	35	Adequate	Adequate	Present	1.2	1356	17	Adequate	Adequate	Present	6	925	28
4	Adequate	Adequate	Present	1.2	1244	37.8	Adequate	Adequate	Present	1.5	950	36.7	NI	NI	NI	NI	NI	NI
5	Adequate	Adequate	Present	9	1119	28	Adequate	Adequate	Present	1.17	1284	25	Adequate	Adequate	Present	9	1097	32
6	Changed	Adequate	Present	1	1044	88	Changed	Adequate	Present	1.21	1097	82	NI	NI	NI	NI	NI	NI
7	Changed	Increased	Present	1.4	959	38.4	Changed	Increased	Present	1.4	1006	30.4	NI	NI	NI	NI	NI	NI
9	Adequate	Adequate	Present	0.6	1369	34.3	Adequate	Adequate	Present	0.4	1022	22.8	Adequate	Adequate	Present	1.4	990	34.4
10	Adequate	Adequate	Present	2.4	1106	93	Adequate	Adequate	Present	1.5	866	54.5	Adequate	Adequate	Present	2.4	990	76
11	Adequate	Adequate	Present	1	1062	89	Adequate	Adequate	Present	0.7	1254	83.2	Adequate	Adequate	Present	0.7	889	66.1
12	Adequate	Adequate	Present	1	1221	83	NA	NA	NA	NA	NA	NA	Adequate	Adequate	Present	1.4	1040	76
14	Adequate	Adequate	Present	0.43	1011	59	NA	NA	NA	NA	NA	NA	Adequate	Adequate	Present	1.4	903	61
15	Changed	Increased	Present	1	914	72	Changed	Increased	Present	0.78	912	78	NI	NI	NI	NI	NI	NI
16	Adequate	Adequate	Present	0.7	1119	79	Adequate	Adequate	Present	1	1200	98	Adequate	Adequate	Present	0.7	1020	79
17	Adequate	Adequate	Present	0.7	688	56	NA	NA	NA	NA	NR	NA	Adequate	Adequate	Present	1	719	39.6
18	Adequate	Adequate	Present	0.78	1110	69	Adequate	Adequate	Present	1.1	1201	56	Adequate	Adequate	Present	1	1350	98
19	Changed	Increased	Present	0.6	780	67	Changed	Increased	Present	0.7	860	30.8	NI	NI	NI	NI	NI	NI
20	Adequate	Adequate	Present	0.8	1127	92	Adequate	Adequate	Present	1.11	1231	69	Adequate	Adequate	Present	1	1131	84
21	Adequate	Adequate	Present	0.7	774	54	Adequate	Adequate	Present	0.72	685	69	NA	NA	NA	NA	NA	NA
22	Adequate	Adequate	Present	0.75	774	59	NA	NA	NA	NA	NA	NA	NA	NA	NA	NA	NA	NA

Child 13 was using a nasogastric tube and child 8 was not evaluated. NA: not evaluated, NI: food supply not introduced, average frequency (Hz), average loudness (dB). Reference values: time (s): 0.8 a 1.2/average frequency (Hz) 800 to 1800/average loudness (dB): 70 a 100. Microcephalic patients are marked in gray.

**Table 4:** Comparison of baseline characteristics and changes in orofacial motricity and saliva and swallowing assessment in children exposed to Zika virus in the intrauterine period in Manaus, Amazonas, Brazil.

Characteristic	Overall, *n* = 22	Oropharyngeal Dysphagia Absent *n* = 16	Oropharyngeal Dysphagia Present *n* = 6	*p* Value ^1^
Microcephaly	5/22 (22.73%)	0/16 (0.00%)	6/6 (100.0%)	<0.001
Sex				0.4
Male	14/22 (63.64%)	9/16 (56.25%)	5/6 (83.33%)	
Age ^2^	35 (5)	35 (4)	35 (6)	0.6
Gestational age				0.6
36	1/22 (4.55%)	1/16 (6.25%)	0/6 (0.00%)	
38	3/22 (13.64%)	2/16 (12.50%)	1/6 (16.67%)	
39	14/22 (63.64%)	9/16 (56.25%)	5/6 (83.33%)	
40	4/22 (18.18%)	4/16 (25.00%)	0/6 (0.00%)	
Birth weight (kg)^2^	2.99 (0.45)	3.05 (0.51)	2.84 (0.19)	0.3
Apgar				0.3
8	4/22 (18.18%)	2/16 (12.50%)	2/6 (33.33%)	
9	18/22 (81.82%)	14/16 (87.50%)	4/6 (66.67%)	
Adequate NHS	19/20 (95.00%)	16/16 (100.00%)	3/4 (75.00%)	0.2
Tongue test changed	1/14 (7.14%)	1/11 (9.09%)	0/3 (0.00%)	>0.9
Breathing and heart problems				0.6
Cardiac	1/22 (4.55%)	0/16 (0.00%)	1/6 (16.67%)	
Respiratory	1/22 (4.55%)	1/16 (6.25%)	0/6 (0.00%)	
Respiratory and cardiac	1/22 (4.55%)	1/16 (6.25%)	0/6 (0.00%)	
Inadequate vocal quality	5/22 (22.73%)	1/16 (6.25%)	4/6 (66.67%)	0.009
Inadequate frequency of swallowing	5/22 (13.64%)	0/16 (0.00%)	5/6 (50.00%)	<0.001
Altered cervical auscultation	3/22 (13.64%)	0/16 (0.00%)	3/6 (50.00%)	0.013
Average time for liquids	1.69 (2.22)	1.90 (2.55)	1.04 (0.30)	0.8
Average frequency for liquids	1.051 (191)	1.072 (198)	988 (172)	0.3
Average loudness for liquids	57 (27)	56 (29)	61 (22)	0.8
Average time for pasty consistencies	1.00 (0.35)	0.94 (0.35)	1.12 (0.36)	0.3
Average frequency for pasty consistencies	1.089 (209)	1.151 (227)	965 (91)	0.075
Average loudness for pasty consistencies	51 (28)	51 (30)	52 (26)	0.8
Average time for solids	2.36 (2.67)	2.36 (2.67)	NA	
Average frequency for solids	1.005 (160)	1.005 (160)	NA	
Average loudness for solids	61 (24)	61 (24)	NA	

^1^ Wilcoxon rank sum exact test; Fisher’s exact test; Wilcoxon rank sum test. ^2^ Median (IQR) [Mean (SD)]. NA: Not avaliable

## Data Availability

All data are contained within the article.

## Supplementary Materials

The following supporting information can be downloaded at: https://www.mdpi.com/article/10.3390/v15122363/s1, Table S1: Neurological characteristics for microcephalic children.; Table S2: Growth velocity in children exposed to the Zika virus in the intrauterine period in Manaus, Ama-zonas, Brazil.
