# Supplementary material for: Clinical and Acoustic Alterations of Swallowing in Children Exposed to Zika Virus during Pregnancy in a Cohort in Amazonas, Brazil: A Case Series Study
Source: Viruses. 2023 Nov 30;15(12):2363. doi: 10.3390/v15122363 (PMC10747239; doi:10.3390/v15122363)
Supplement: Supplementary file 1 [file viruses-15-02363-s001.zip › viruses-2641401-supple 1.pdf]

Supplementary table 1: Neurological characteristics for microcephalic children.

ID	Neurological characteristic
4	Congenital microcephaly, epileptic seizures starting at 4 months of age, West syndrome, global developmental delay (cognition, language, motor, social), deficit in muscle strength with tetraparesis, spasticity, increased osteotendinous reflex response.<br>Skull tomography: lissencephaly, intracranial calcifications at the corticomedullary junction, dilation of the ventricular system and cerebral atrophy
7	Microcephaly crises, global developmental delay (cognition, language, motor, social), deficit in muscle strength with tetraparesis, spasticity, increased osteotendinous reflex response.
19	Congenital microcephaly, epileptic seizures starting in the neonatal period, global developmental delay (cognition, language, motor, social), deficit in muscle strength with tetraparesis, spasticity, increased osteotendinous reflex response.<br>Skull tomography lissencephaly, intracranial calcifications, dilatation of the ventricular system and cerebral atrophy

Supplementary table 2: Growth velocity in children exposed to the Zika virus in the intrauterine period in Manaus, Amazonas, Brazil.

ID	Growth speed
1	ND
2	Proper
3	Proper
4	Proper
5	Proper
6	ND
7	ND, appropriate weight
8	ND
9	Inappropriate
10	Proper
11	Proper
12	ND
13	Proper
14	Inappropriate
15	ND
16	Proper
17	Proper
18	Proper
19	Proper
20	ND
21	Proper
22	Proper

ID: identification and ND: Not determined. Microcephalic patients are marked in gray.
